# Regular Patterns in Cerebellar Purkinje Cell Simple Spike Trains

**DOI:** 10.1371/journal.pone.0000485

**Published:** 2007-05-30

**Authors:** Soon-Lim Shin, Freek E. Hoebeek, Martijn Schonewille, Chris I. De Zeeuw, Ad Aertsen, Erik De Schutter

**Affiliations:** 1 Theoretical Neurobiology, University of Antwerp, Antwerp, Belgium; 2 Department of Neuroscience, Erasmus MC, Rotterdam, The Netherlands; 3 Neurobiology and Biophysics, Faculty of Biology and Bernstein Center for Computational Neuroscience, Albert-Ludwigs-University, Freiburg, Germany; 4 Computational Neuroscience Unit, Okinawa Institute of Science and Technology, Okinawa, Japan; Duke University, United States of America

## Abstract

**Background:**

Cerebellar Purkinje cells (PC) in vivo are commonly reported to generate irregular spike trains, documented by high coefficients of variation of interspike-intervals (ISI). In strong contrast, they fire very regularly in the in vitro slice preparation. We studied the nature of this difference in firing properties by focusing on short-term variability and its dependence on behavioral state.

**Methodology/Principal Findings:**

Using an analysis based on CV_2_ values, we could isolate precise regular spiking patterns, lasting up to hundreds of milliseconds, in PC simple spike trains recorded in both anesthetized and awake rodents. Regular spike patterns, defined by low variability of successive ISIs, comprised over half of the spikes, showed a wide range of mean ISIs, and were affected by behavioral state and tactile stimulation. Interestingly, regular patterns often coincided in nearby Purkinje cells without precise synchronization of individual spikes. Regular patterns exclusively appeared during the up state of the PC membrane potential, while single ISIs occurred both during up and down states. Possible functional consequences of regular spike patterns were investigated by modeling the synaptic conductance in neurons of the deep cerebellar nuclei (DCN). Simulations showed that these regular patterns caused epochs of relatively constant synaptic conductance in DCN neurons.

**Conclusions/Significance:**

Our findings indicate that the apparent irregularity in cerebellar PC simple spike trains in vivo is most likely caused by mixing of different regular spike patterns, separated by single long intervals, over time. We propose that PCs may signal information, at least in part, in regular spike patterns to downstream DCN neurons.

## Introduction

The cerebellum is crucial for the precise temporal control of motor related tasks [1] and conditioned behaviors [2]. Yet, it is not clear how the cerebellum may signal precise timing at the cellular level. Prior studies of spike time coding in the cerebellum have focused on the discharge of Purkinje cells (PCs), which form the sole output of cerebellar cortex. However, thus far these studies only considered mean firing rates of the simple spikes (SS) [3], [4] or complex spikes (CS) [5], [6]. Little attention has been paid to their fine-temporal structure, even though spike timing may encode additional information in other systems [7]–[12]. In fact, for two different strains of ataxic mice with mutations of voltage-gated calcium channels expressed in PCs it was recently reported that PCs show increased irregularity of firing [13], [14].

A common measure to characterize the temporal structure of spike trains is the coefficient of variation (CV) of the interspike intervals (ISIs). The CV of SS firing of PCs recorded *in vivo* is reported to be quite high [15], [16]: close to or even higher than 1, the CV of a Poisson process. Conversely, PCs in the *in vitro* slice preparation fire very regularly [17], [18]. To test whether this difference in firing properties is as large as is commonly assumed and to investigate its possible functional importance, we analyzed the fine-temporal structure of SS trains in different preparations and behavioral states in more detail, focusing on the short-term variability.

## Materials and Methods

### Recordings

#### Rats

Sprague-Dawley rats (n = 26, 300–500 g, Iffa Credo, Brussels, Belgium) were anesthetized with a mixture of ketamine HCl (75 mg/kg; Ketalar, Parke-Davis, Warner Lambert Manufacturing, Dublin, Ireland) and xylazine HCl (3.9 mg/kg; Rompun, Bayer, Leverkusen, Germany) in normal saline (0.9% NaCl, Baxter, Lessine, Belgium) by intraperitoneal injection. A craniotomy exposing Crus I and II of the left cerebellar hemisphere was performed [16]. Supplemental doses (one-third initial dose) were given intramuscularly to maintain deep anesthesia as evidenced by the lack of a pinch withdrawal reflex and/or lack of whisking. Forty eight single unit recordings were made in the cerebellar cortex with tungsten microelectrodes (impedance ∼10 MOhm, FHC, Bowdoinham, ME). Signals were filtered and amplified (bandpass = 0.5–9 kHz; gain = 5,000–10,000) using a multichannel neuronal acquisition processor (Plexon Inc., Austin, TX) and collected spike trains were analyzed off-line using NEX (Plexon Inc.). After recordings of spontaneous activity, 12 stimulus-evoked responses were recorded in 10 rats. Perioral receptive fields were explored as reported elsewhere [16]. The punctate stimulus was applied at a rate of 0.5 Hz. In a separate series of experiments reported in more detail in [19], 8 transverse pairs of nearby PCs were recorded using similar procedures. Electric lesions were made after recordings to measure the distance between pairs and the distance between the centers of lesions was measured. In the context of this paper, the data from all these experiments were re-analyzed. All experimental methods were approved by the University of Antwerp and conformed to European Union guidelines.

#### Mice

Extracellular activity was recorded in the cerebellar flocculus and paramedian lobule using glass micropipettes filled with 2 M NaCl (tip diameter: 2–5 mm; impedance: 2.5 MΩ at 1 kHz) in either restrained awake or anesthetized (with mixture of ketamine (50 mg/kg) and xylazine (10 mg/kg)) C57BL/6 mice. The electrode tip was positioned on the cerebellar surface under visual guidance (Olympus VS-IV; Olympus Optical, Tokyo, Japan) using a micromanipulator (David Kopf Instruments, Tujunga, CA) and moved downward by a hydraulic microdrive (Trent Wells) equipped with a stepping motor (TL Elektronik SMS 87). The electrode signal was amplified and filtered (bandwidth 10–6000 Hz; Dagan 2400; Dagan, Minneapolis, MN) and sampled at 12.5 kHz (CED 1401plus, Spike2, Cambridge, UK). Single-unit PCs were identified on-line by the presence of a brief pause in simple spikes after the complex spike. In the off-line analysis, SSs and CSs were detected and discriminated using custom-made software implemented in Matlab (Mathworks, Natick, MA). All experimental methods were approved by Erasmus MC in Rotterdam, and conformed to European Union guidelines.

### Spike timing analysis

#### Data analysis of extracellular recordings

Recordings were 83 to 1202 sec long and comprised 1,328 to 62,371 spikes. Analysis was carried out using Matlab and Excel (Microsoft). Short-term regularity was measured with CV_2_ = 2|ISI_n+1_−ISI_n_|/(ISI_n+1_+ISI_n_) [20]. The number of regular patterns was measured using a threshold value for CV_2_ ranging from 0 to 2 with an increment of 0.02. In each PC the numbers were normalized by the maximum number of patterns to avoid an influence of the difference between firing rates. The strength of synchronization of regular patterns was measured using a standard score, the Z score of the amplitude of the central peak of the cross-correlogram [21]: Z = (N_c_−N_e_)/(SD_e_), with N_c_ the number of spikes in the central peak (bin = 5 ms), N_e_ the mean number of spikes in a 2 sec window between −1 and 1 sec, and SD_e_ its standard deviation. To determine whether the observed synchronization (Z score of 3 or higher) reflected spike to spike precise synchronization or rather co-modulation of firing rates [22], [23], simulated spike trains were generated by randomly shuffling the ISIs within blocks of 5 consecutive ISIs and the correlation analysis repeated for the shuffled spike trains. The correlation between CSs and patterns were analyzed in 32 PCs where CSs were well isolated. If the duration from the ends of patterns to CSs was longer than 1.2 times the pattern mean ISI, it was considered longer than the pattern mean ISI.

#### Data analysis of whole-cell clamp recordings

Three membrane potential traces recorded from 3 different anesthetized rats were analyzed to investigate the relation between spike patterns and the membrane potential [24]. The sampling frequencies of the original recordings (50, 50, and 20 kHz) were sampled down to 10 kHz without loosing discriminative power. Spikes were sorted by setting thresholds at −38 mV, −42 mV, and −32 mV in cell1, cell2 and cell3 respectively. Spikes were further sorted as either CS or SS by checking the mean membrane potential between 2 and 4 ms after a spike. If the mean membrane potential was higher than −38 mV, −38 mV, and −40 mV in cell 1, 2, and 3 respectively, the spike was sorted as a CS. Mean firing rate of SS (CS) was 12.8±5.2 Hz (1.1±0.4 Hz). The threshold to distinguish UP from DOWN states was set to −55 mV. Regular patterns and single intervals were isolated as in the extracellular recordings. Data are represented as mean±s.e.m., unless otherwise stated. All p-values refer to Student's paired or unpaired t-test, unless otherwise specified.

### Stochastic Modeling of Poisson processes

Both spontaneous and evoked spike trains were modeled using an inhomogeneous Poisson process (see also [25]) with 2 ms of dead time (which is equal to the detection window used in the electrophysiological recordings). The probability density of the process can be described as 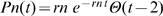, where t denotes the time elapsed since the last spike, r_n_ is the mean firing rate at the n-th ISI, estimated by 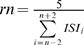 where n = 3, 4, …, number of spikes-2, *Θ* (t−2) is Heaviside function standing for 1 only when t is 2 ms or larger. The mean firing rates of realized spike trains (51.7±2.4 Hz, N = 92) were statistically similar to those of recorded SS trains (51.7±2.4 Hz, N = 92, p>0.6). For evoked spike trains, firing rate was estimated from the rate distribution around stimulation time (bin size: 1 ms, lag = 1 s). Based on these estimates, model spike times were created trial by trial. Then, a final spike train was constructed by concatenating spike times in consecutive trials. Simulations were performed using Matlab.

### Synaptic Conductance Modeling

**Figure 1:**
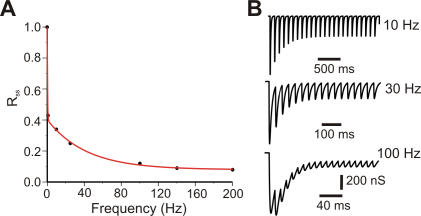
Simulation of PC to DCN synaptic conductance. (A) Saturating level of release probability (Rss) taken from Pedroarena and Schwarz (2003) could be modeled with a double exponential function (red line, see Materials and Methods for details). (B) Simulated synaptic conductance profiles in response to 10, 30 and 100 Hz PC firing, respectively. These results should be compared to Figure 7A of Pedroarena and Schwarz (2003).

The dynamics of multiple pulse depression of the synapse between PCs and DCN neurons were described previously [26], [27]. The data reported in these papers are quantitatively different, even though they report the same phenomena, probably due to different experimental conditions (e.g. recording temperature). Our phenomenological model is based on the Pedroarena and Schwarz study [26], because it measured multiple pulse depression at more frequencies over a large range (from 1 to 200 Hz). The depression is assumed to be caused by changes of the presynaptic release probability (R). We fitted a deterministic model for R to the multiple pulse depression data. This deterministic approach is justified because the large number of release sites from which transmitter can reach all receptors [28] makes synaptic failures unlikely. We fitted equations for the steady state level of release probability *R_ss_* and the time constant of depression *τ* to the data:
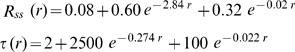
where *r* is the instantaneous firing rate computed as the inverse of the last interspike interval. *R_ss_* and τ are updated at the time of occurrence of each spike *n* and *R_n_* is then computed as:
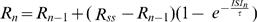
with *R_n−1_* is the release probability computed at the previous spike time, *ISI_n_* is the interspike interval between the current spike and the previous spike. See Figure 1 for the accuracy of the fit of our model to the experimental data.

Synaptic conductance (*G_syn_*) during spiking was modeled by a double exponential function multiplied by *R_n_* and calculated over 200 ms with a resolution of 0.1 ms following each spike:
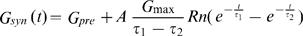
Here, *G_pre_* is the synaptic conductance caused by previous spikes, *A* = 15.5 is a constant to scale the maximum conductance to the experimental value of *G_max_* (11.7 nS), τ*_1_* is 12 ms, and τ*_2_* is 1.2 ms. *G_max_*, τ*_1_*, and τ*_2_*, were chosen to fit the multiple pulse depression traces shown in Figure 7 of Pedroarena et al [26]. The multiple pulse depression following 10, 30 and 100 Hz stimulation of the PC is shown in Figure 1.

## Results

### Simple spike trains contain precise regular spiking patterns

**Figure 2:**
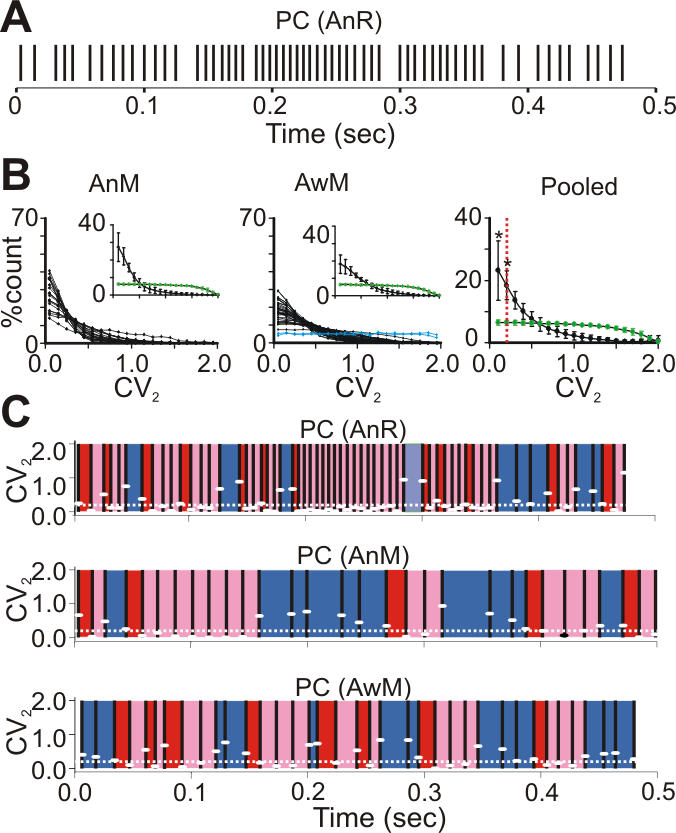
Regular patterns in cerebellar Purkinje cell simple spike trains. (A) Raster plot of PC SS in an anesthetized rat (AnR). (B) CV_2_ distributions of SS trains recorded from anesthetized mice (AnM, left), awake mice (AwM, middle, blue: neurons in cerebral motor cortex), and mean of 92 CV_2_ distributions (Pooled, right) which were significantly different from those of inhomogeneous Poisson processes with similarly modulated firing rates (p<0.05, χ^2^ test; *: p<0.001, χ^2^ goodness of fit residual test; red line: CV_2_ = 0.2). Insets and right panel: mean±s.e.m. (black: PC, green: inhomogeneous Poisson process) (C) Extracting regular spiking patterns by setting CV_2_ threshold at 0.2 (white dotted lines). White dashes: CV_2_ values calculated from the two surrounding ISIs, red: first ISI of regular patterns, pink: successive ISIs in regular patterns, dark blue: ISIs not belonging to a regular pattern).

We analyzed spontaneous PC activity in 3 data sets: recordings from the cerebellar hemisphere of anesthetized rats (AnR, n = 48) and from the flocculus or paramedian lobule of anesthetized (AnM, n = 21) and awake (AwM, n = 37) mice. Firing rates were similar for all data sets (Table 1). As expected, CVs of the spike trains were high: 3.93±0.49 (AnR), 1.74±0.47 (AnM) and 1.39±0.38 (AwM), consistent with previous reports [6], [16] and suggestive of highly irregular firing *in vivo*. Nevertheless, careful visual inspection of the individual spike trains revealed clear patterns of regular firing (Figure 2A).

**Table 1:** Summary of spontaneous simple spike firing properties of all PCs reported in this study.

	Number of PCs	Mean firing rate	%Long ISI (ISI>1 s)	CV	Mean CV_2_	Maximum pattern size
Anesthetized rats	48	45.5±4.1	0.41±0.11	3.93±0.49	0.51±0.03	28.9±4.4
Awake mice	37	51.0±2.7 (p>0.2)	0.01±0.00 (p<0.001)	1.39±0.38 (p<10^−4^)	0.39±0.02 (p<0.001)	13.0±0.6 (p<0.001)
Anesthetized mice	21	49.8±3.6 (p>0.4, p^*^>0.7)	0.02±0.01 (p<0.001, p^*^>0.1)	1.74±0.47 (p<0.05, p^*^>0.4)	0.30±0.02 (p<10^−6^, p^*^<0.05)	24.9±2.8 (p>0.4, p^*^<0.001)

p: comparison to anesthetized rats, p^*^: comparison to awake mice, Student t test.

To characterize these patterns we used a short range measure which compares two adjacent ISIs, i.e. the CV_2_ (cf. Materials and Methods; [20]). Surprisingly, we found that in all data sets the mean CV_2_ was low (AnR: 0.51±0.03, AnM: 0.30±0.02, AwM: 0.39±0.02), suggestive of much more regular firing at short time scales. In fact, most PCs showed a skewed CV_2_ distribution, with a high proportion of low CV_2_ values (Figure 2B), indicating the presence of regularity in spiking patterns. This was in clear contrast to spontaneous spiking of neocortical neurons, which showed uniform CV_2_ distributions as previously reported [20] (Figure 2B, blue) and which are similar to realizations of inhomogeneous Poisson processes (insets in Figure 2B, green).

We studied the properties of regular spiking patterns in PCs whose CV_2_ distribution was significantly different from rate modulated Poisson (AnR: n = 38, AnM: n = 21, AwM: n = 33, p<0.05, χ^2^ test) in more detail. Their pooled CV_2_ distribution showed significantly more CV_2_ values of 0.2 or lower (p<0.001, χ^2^ goodness of fit residual test) than the corresponding Poisson processes did (Figure 2B, rightmost panel). To isolate the regular spiking patterns in individual spike trains, we applied a threshold of 0.2 on the measured CV_2_ values as illustrated in Figure 2C. Whenever the CV_2_ value was below or equal to threshold (white dotted line), the associated two ISIs were considered part of a regular pattern (pink). If the next ISI also had a CV_2_ value below or equal to threshold, it was included into the pattern (pink); if not, this next ISI was either single (i.e. not belonging to a pattern; blue) or the start of a new pattern (provided the next CV_2_ value was again below or equal to threshold; red).

**Figure 3:**
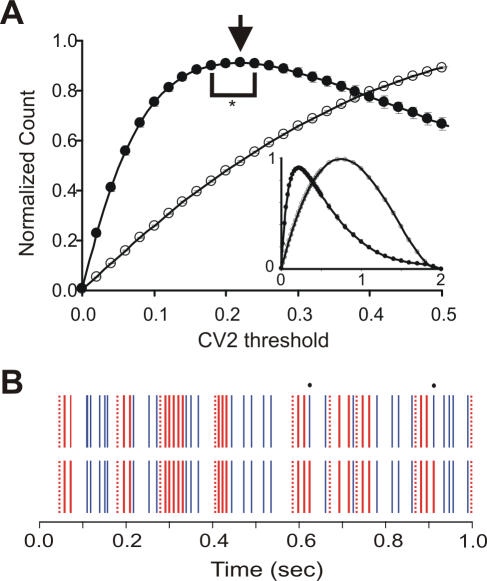
Effect of CV_2_ threshold on patterns. (A) Mean (± s.e.m.) of normalized number of patterns in spike trains classified with different values of the CV_2_ threshold, ranging from 0 to 0.5, in 92 PCs (filled circles) and in simulated spike trains from Poisson processes with similar firing rate profiles as in the PCs (open circles). Arrow: maximum number of patterns, *: range where there was no statistical difference (p>0.05). Inset: same distribution but for all possible thresholds. (B) Raster plots with indication of the spike timings belonging to patterns (red dotted lines: start of patterns, red solid lines: following spikes in each pattern) and singles (blue). Black dots: difference in classified patterns when threshold was 0.2 (upper trace) and 0.24 (lower trace).

With this procedure, 57% (AnR), 67% (AnM) and 54% (AwM) of ISIs belonged to regular patterns. To verify the effect of the statistically defined CV_2_ threshold of 0.2, we compared the number of patterns extracted using different thresholds (Figure 3). Thresholds in the range 0.18–0.24 generated statistically similar number of patterns as a threshold of 0.22, which generated the maximum number of patterns (n = 92, p>0.1).

**Figure 4:**
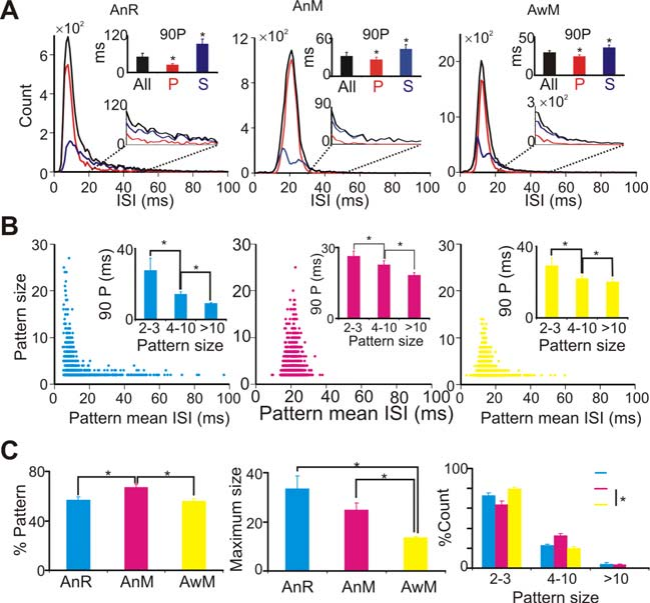
Characteristics of regular spike patterns. (A) ISI distribution of overall ISIs (black), patterns (red) and singles (blue) from a representative sample PC spike train of AnR (left), AnM (middle) and AwM (right). Insets: magnified plot of indicated area (lower) and 90 P (90 percentile, upper) of each population, *: p<0.01, Student t test. (B) The relation between pattern mean ISI and pattern size in AnR (left, cyan), AnM (middle, magenta) and AwM (right, yellow). Insets: maximum pattern mean ISI (90 percentile) of different pattern sizes. *: p<0.001, Wilcoxon signed rank test. (C) Percentage ISIs belonging to patterns (upper, *: p<0.001, Student t test), Average maximum pattern size (middle, *: p<0.001, Student t test), and Pattern size distribution (lower, p<0.05, χ^2^ test). Cyan: AnR, magenta: AnM, yellow: AwM.

The mean ISIs of patterns were not uniformly distributed. Most of the pattern ISIs were relatively short, so that the peaks of the overall ISI distributions mostly consisted of regular patterns, while their tails comprised only single ISIs. As a result, the 90 percentile of ISI for patterns was significantly shorter than that of singles (Figure 4A).

### Characteristics of regular spiking patterns change with behavioral state

Regular patterns can be characterized by two parameters: pattern size, defined as the number of ISIs in the pattern, and pattern mean ISI. Examples of the distribution of these two parameters are shown in Figure 4B. Observe that short patterns occurred with a wide range of mean ISIs, whereas long patterns contained only short ISIs (insets), though not the shortest. The wide range of pattern mean ISIs and the fact that the fraction of pattern spikes was only weakly (rats: linear correlation R^2^ = 0.371 compared to a R^2^ = 0.666 for 92 inhomogeneous Poisson processes) or not (mice: R^2^<0.1) dependent on the mean firing rates of PCs make it unlikely that regular patterns were caused by refractoriness.

Pattern sizes showed a wide distribution. On average, 72% of patterns comprised only 2–3 ISIs, but many patterns were much longer (Figure 4B and 4C), lasting 45.0±3.5, 76.5±6.3, and 52.5±3.1 ms for AnR, AnM, and AwM, respectively. The size of patterns depended on the CV_2_ threshold used, but this did not affect the pattern mean ISI much (data not shown). Interestingly, we found a significant difference in the proportion of long patterns between anesthetized and awake rodents. In anesthetized rodents, 4.1±1.4% (AnR) and 3.5±0.8% (AnM) of patterns contained more than 10 ISIs, while in awake rodents (AwM) only 0.4±0.1% did (p<0.01), with maximum pattern sizes of 182, 61 and 21 ISIs, respectively (Figure 4C). This significant difference between pattern sizes of awake vs. anesthetized rodents indicates that regular patterns may be influenced by the behavioral state of the animal. Indeed, if regular spiking patterns were a specific signal by which PCs transmit information, one would predict faster changes in this signal, i.e. shorter patterns associated with a wider range of pattern mean ISIs, in awake, active animals than in anesthetized ones.

### Simulation of the effects of regular spiking patterns on target neurons

**Figure 5:**
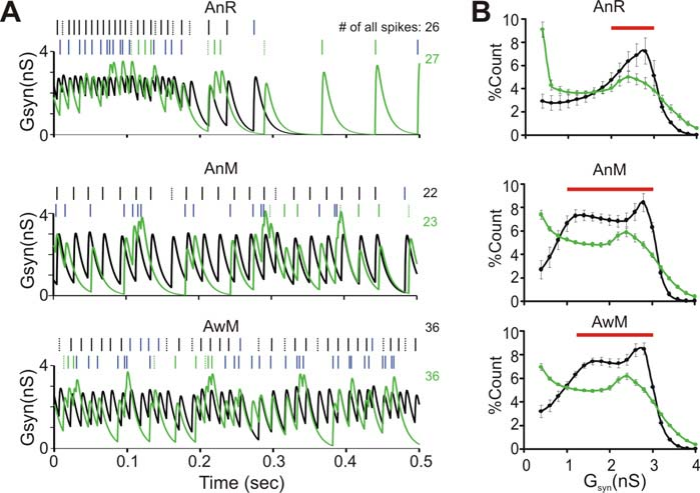
Simulated synaptic conductance in PC to DCN synapse caused by spontaneous PC spiking. (A) A representative example of the simulated synaptic conductance (G_syn_) induced by PC (black) of AnR (upper panel), AnM (middle panel) and AwM (lower panel), and by corresponding realizations of an inhomogeneous Poisson process (green). Rasters: spikes belonging to patterns (black and green dotted lines: start of patterns, black and green solid lines: following spikes in patterns, blue lines: singles), numbers: number of all spikes in the 500 ms window. (B) Distribution of G_syn_ values for PCs (black) compared to Poisson processes (green). Bin = 0.2 nS. Red bar: bins where PCs contained significantly more G_syn_ values. p<0.05.

PCs inhibit neurons in the downstream DCN; any information transmitted by regular PC spike patterns will be decoded at that level. PC synapses onto DCN neurons show fast synaptic depression [26], [27], a property that is known to endow synapses with low-pass filtering properties [29]. We developed a phenomenological model to reproduce the previously reported frequency-dependent depression of this synapse [26] (cf. Materials and Methods), allowing us to predict the effects of regular PC spike patterns on synaptic conductance (G_syn_) in DCN neurons. Specifically, we used this model to compare the G_syn_ evoked by recorded SS trains with that of simulated spike trains generated by inhomogeneous Poisson processes of the same modulated firing rates. Such Poisson processes have far fewer regular patterns: only 20% of ISIs belonged to patterns, 80% of which were of size 2 (cf. Fig. 2B). A representative example of conductance traces (Figure 5A) demonstrates that the long regular patterns in SS trains induced epochs with little fluctuation of G_syn_, while Poisson spike trains generated much more variable G_syn_ (Figure 5A, green). In almost all cases, the distribution of G_syn_ of Poisson spike trains was significantly different from that of real SS trains (Figure 5B; Kolmogorov-Smirnov test, p<0.05, bin 0.01 s, AnR 35/38 cells; AnM 33/33; AwM 20/21). Thus, the distribution of G_syn_ of PCs was mostly confined to a narrow range of values as is also evident from its CV, which was significantly lower for PCs (0.53±0.03, 0.47±0.03 and 0.49±0.03 for AnR, AnM and AwM, respectively) than for simulated spike trains from Poisson processes (0.66±0.05, 0.75±0.04, and 0.71±0.03, p<0.04).

### Spikes of regular patterns are correlated, but not precisely synchronized

**Figure 6:**
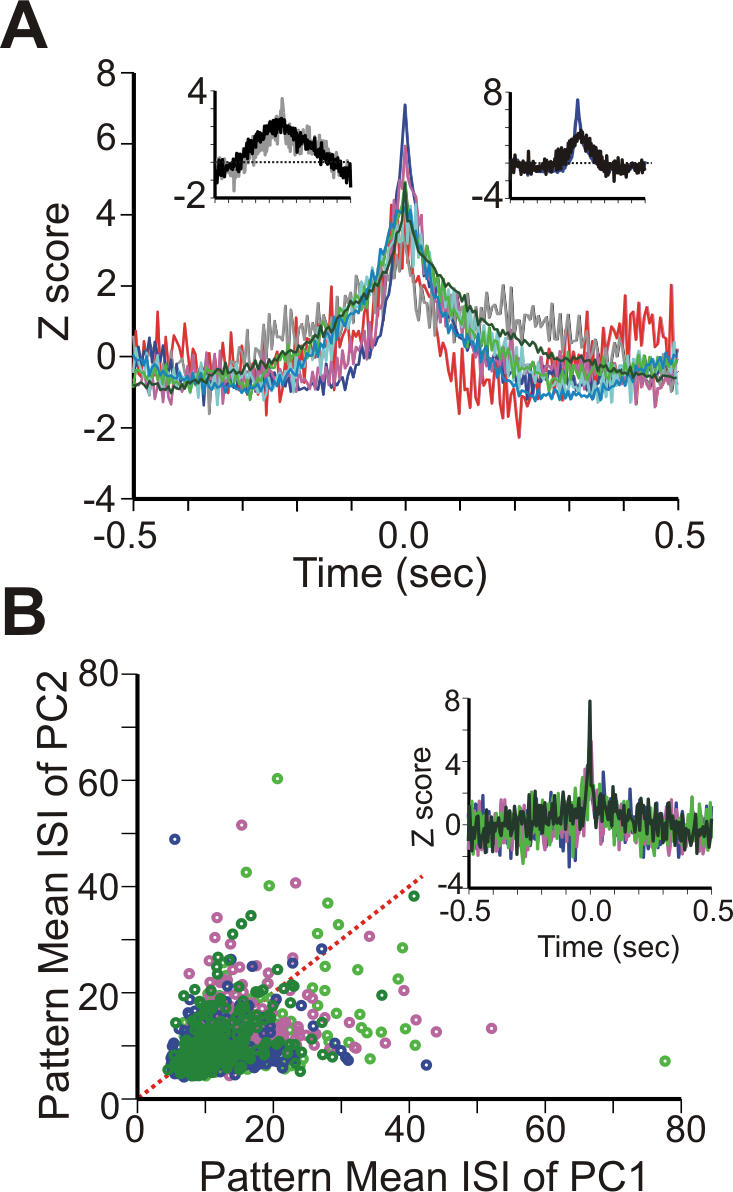
Coincident patterns in nearby PC pairs in AnR. (A) Eight cross-correlograms of timings of spikes belonging to regular patterns extracted from recordings of nearby PC pairs, with each pair colored differently. Insets: cross-correlograms of the shuffled spike trains of two pairs (black) superimposed on original cross-correlogram of patterns (blue and gray: pairs showing strongest and weakest synchronization respectively). (B) The relation of pattern mean ISIs in 4 pairs in which pattern starts coincided significantly (inset: cross-correlograms of the first spikes of regular patterns in the 4 pairs). Red dotted line: diagonal.

In rats, each DCN neuron receives inhibition from 100 [30] up to 1000 [31] PCs. Anatomically, though, it is not clear whether all converging inputs are active at the same time. This convergence raises the question whether regular patterns in the afferent PCs coincide in time, causing periodic ripples in G_syn_ during their occurrence, or whether they are asynchronous, rendering G_syn_ more constant. We studied the correlation in time of regular patterns in 8 simultaneously recorded transverse pairs of PCs in AnR, separated by 69.8±9.4 µm (range: 50–100 µm). We found that the spikes belonging to patterns revealed central peaks in the cross-correlogram. These central peaks reflected significant synchronization as their Z scores were higher than 3 (Figure 6A, z = 5.0±0.4), but they were quite broad (full width at half peak (HW) = 70±8.6 ms). No synchronization was observed in pairs of PCs on the same parallel fiber beam separated by more than 0.5 mm (n = 20, data not shown). We investigated several mechanisms that could explain the broad width of the central peaks. There was no significantly relation between HW and the mean duration of patterns (R^2^<0.0001). Broad peaks in cross-correlograms are often caused by firing rate co-modulation [23], implying that patterns would coincide because they occur more often during increased firing rates (Figure 4A). As properly shuffled spike trains (cf. Materials and Methods) overlapped the broad peaks largely (Figure 6A, insets), the central peaks observed can indeed be largely explained by firing rate co-modulation. In addition, we found in four of the pairs a significant, and much more precise, correlation of the start of patterns (z = 5.6±0.9, HW = 16.3±2.4 ms, Figure 6B inset). But although patterns in these four pairs started together, their mean ISIs were independent of each other (R^2^ = 0.17±0.03, Figure 6B). We conclude that while for a fraction of patterns the start was precisely synchronized, overall pattern spikes were not precisely synchronized but tended to co-occur in a loose manner because of firing rate co-modulation.

Despite our lack of knowledge about detailed convergence patterns of PCs onto DCN neurons, convergence is more likely for adjacent pairs, where we observed coincident patterns, than for distant pairs which did not show synchronization. In the case of coincident converging patterns, the lack of spike synchronization caused by their different spike frequencies will further reduce the variability of their combined G_syn_ [32]. Otherwise, the averaged G_syn_ of perfectly synchronized PCs would have the same variability as that caused by single PCs. A similar reduction of variability also occurs in completely irregular spike trains generated by Poisson processes [32], but only when there are many more active convergent inputs.

### Tactile stimulation increases regularity of spiking

**Figure 7:**
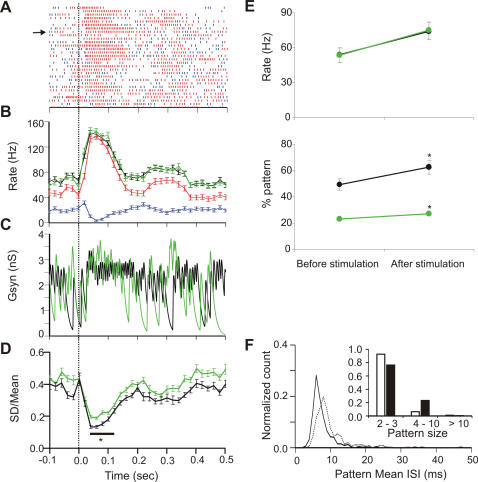
A representative example of regular patterns in tactile stimulus evoked PC SS responses. (A) Peri-event raster plot of patterns (red) and singles (blue) during tactile stimulation in AnR. (B) Mean rate (± s.e.m.) of overall spikes (black), realization of Poisson process (green), pattern spikes (red) and singles (blue). Bin = 20 ms. (C) Simulated G_syn_ for the trial indicated by arrow in (A) (bin = 1 ms). (D) CV (SD/Mean) of simulated G_syn_ (*: p<0.001, Student t test, bin 20 ms). Black dotted line: stimulation time. (E) Mean firing rate (upper panel) and percent ISIs belonging to regular patterns (lower panel) in 200 ms before and after stimulation (upper panel) of simulated spike trains from inhomogeneous Poisson process (green) and from recorded PCs (black). *: p<0.005, Wilcoxon signed ranks test. (F) Pattern mean ISI distribution before (dotted line) and after (solid line) tactile stimulation. Inset: Pattern size distribution before (open) and after (filled) stimulation.

Next, the effect of sensory stimulation on regular patterns in the SS response was investigated. To this end, we analyzed responses to tactile stimulation in AnR (Figure 7) (n = 12). Typically, PCs responded after a short delay with a significant increase in SS firing rate in a 200 ms window, from 53.8±6.2 Hz to 74.2±7.3 Hz (p<0.003, Wilcoxon signed ranks test), as reported elsewhere [33]. In the same window, we also found a significantly increased proportion of ISIs belonging to regular patterns, from 49.3±4.5% to 62.5±5.1% (p<0.005, Wilcoxon signed ranks test). Spike trains always become more regular at high firing rates because spikes cannot fire arbitrarily close together, due to the refractory period. Indeed, simulated Poisson processes with refractory period that show similar rate changes (p>0.2; before: 53.3±6.3 Hz, after: 75.0±7.5 Hz; Figure 7E upper panel) as the experimental data revealed a slight but statistically significant increase of the fraction of ISIs belonging to regular patterns (p<.005; before: 23.1±0.7%, after: 27.1±0.8%; Figure 7E lower panel). However, this increase was proportionally (18±2%) much smaller than the increase in PCs (29±3%) (p<0.03, Wilcoxon signed ranks test). We conclude that the increase in patterns in PCs was larger than expected from only the firing rate increase. Regular patterns following stimulation were also faster and lasted longer than before stimulation (Figure 7F).

To estimate the effect of this change of SS patterns on downstream DCN neurons, we again computed the predicted G_syn_ and compared these with the results obtained from spike trains realized from inhomogeneous Poisson processes (Figure 7C). The real SS train induced a steady current of about 4.5 nS, while the Poisson process produced a highly variable G_syn_, despite the similar modulations of firing rates. The CV of G_syn_ induced by the real SS train dropped significantly (p<0.001) during a period of 116.7±19.0 ms after stimulus onset compared to the effect of Poisson spike trains (Figure 7D). This indicates that tactile stimulation further reduced the variability of G_syn_ in DCN neurons by an increased regularity of PC spike timing.

### Regular patterns and singles in relation to the PC membrane potential

**Figure 8:**
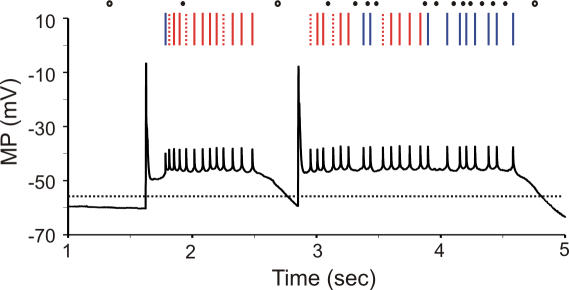
Regular patterns and singles related to the membrane potential (MP). Dendritic patch-clamp recording of PC in anesthetized rat (data from Loewenstein et al. 2005). Voltage trace: large spikes are complex spikes, small ones are simple spikes. Dotted black line: threshold to define up and down-states (MP = −55 mV). Raster plot at top: simple spikes were sorted as either pattern spikes (dotted red lines: start of patterns, solid red lines: following spikes in each pattern) or single spikes (blue lines). All patterns were during up-state, but singles occurred both during up (filled circles) and down (open circles) states.

It has been shown that the membrane potential of PCs in anesthetized animals can be bistable, showing up and down-states [24]. Although PCs in awake behaving animals probably operate predominantly in the up-state [34] and regular patterns in our data tended to last much shorter than the reported duration of up-states in the anesthetized preparation [24], the occurrence of patterns might in principle be related to the state of the membrane potential. We therefore applied our analysis method to whole-cell clamp recordings from PCs of anesthetized rats in vivo (data obtained from Loewenstein et al., 2005; Figure 8). As expected, patterns occurred only during up-states (Figure 8, red spikes), but a single up-state typically consisted of several patterns (4.8±1.1 for the recording shown in Figure 8, 3.8±0.3 for all recordings). Singles could occur during both up (92.1±3.7%; Figure 8, filled dots) or down (7.9±3.7%, Figure 8, open dots) states. Thus, the classification of SSs developed in this study allows for a subcategorization of spikes occurring during the up-state which may be relevant at short time scales.

CSs may toggle transitions between up and down-states [24]. This is also the case for start of the two up-states shown in Figure 8. We found that, except for patterns occurring at state transitions, the start or end of patterns was not related to CS firing. This is confirmed by the much higher frequency of starts of patterns (7.42±4.30 Hz, AnR, n = 32) than of CSs (0.72±0.05 Hz).

## Discussion

Taken together, our main findings indicate that (1) interesting fine-temporal properties of neuronal responses may be uncovered by analyzing regular pattern structure on a single trial basis; (2) PC simple spike trains contain distinctly more spike timing regularities than hitherto known; (3) the high CV in *in vivo* recordings is most likely caused by mixing of different regular spiking patterns, separated by single, typically longer, ISIs; (4) the onset of patterns can be synchronized in nearby PCs, but their member spikes are not synchronized; (5) most regular patterns are not influenced by complex spikes; (6) regular pattern properties change with behavioral state and tactile stimulation; and (7) regular patterns may cause epochs of close to constant synaptic conductance in downstream DCN neurons.

Our extracellular recordings do not provide conclusive evidence on the mechanisms causing regular patterns. However, as PCs fire highly regularly in slice preparations in which their synaptic inputs are blocked [17], [18] and since they show increased irregularity following mutations of their voltage gated Ca^2+^ channels [13], the endogenous properties of PCs are likely to contribute to their regularity of firing. However, the current observation that most patterns occur within the up-state, combined with the finding that PCs in awake behaving animals probably operate predominantly in the up-state [34] suggests that additional mechanisms such as short-term and long-term synaptic processes probably also play a role in controlling the start and end of a regular pattern, as well as its mean ISI. As CSs have little effect on patterns, it is most likely that parallel fiber inputs combined with molecular layer inhibition control the pattern properties. Furthermore, synaptic plasticity can adapt the effect of both the excitatory parallel fiber inputs and the inhibitory input from the basket cells and stellate cells on the SS patterns [35], [36]. Such mechanisms could explain why the onset of patterns was synchronized in only a subset of nearby PCs, and why even in those cases the pattern mean ISIs were different.

The regular patterns discovered in this study comprised a large part of the simple spike trains and were shown to be modulated by behavioral state and stimulation, suggesting that they may have functional significance. Our simulations of the effect of regular patterns on G_syn_ in downstream DCN neurons indicate that they keep inhibitory conductance fairly constant. The interaction between regular patterns and G_syn_ may provide an explanation of why these synapses depress so strongly [26], [27] and forms the basis for our hypothesis on the function of regular patterns. It is generally assumed that cerebellar learning through induction of long-term depression at the parallel fiber to PC synapse leads to disinhibition of DCN neurons [35], [37]. In addition, DCN neurons respond strongly to disinhibition because of their post-inhibitory rebound spike [38], which may form a powerful timing signal [2], [39]. Correspondingly, the activity of DCN neurons in adult rodents consists of pauses, most likely caused by PC inhibition, mixed with transient periods of fast bursting [40]. The effectiveness of disinhibition to create a rebound spike depends on the synchronicity of the disinhibition, which we recently demonstrated to be significant among nearby PCs [19], and on the level of preceding inhibition. Because the inactivation of calcium channels expressed in the DCN neurons is strongly voltage dependent in the relevant potential range [41], these channels are very sensitive to even small changes in inhibitory input. Consequently, the level of inhibition preceding the rebound spike exerts a very strong effect on the amplitude of the rebound spike [42].

We hypothesize that regular patterns encode a specific level of inhibition in their firing rate and, as such, approximate a perfect firing rate code [43], which should be completely regular. When regular patterns from convergent PCs coincide, the summed inhibition will be relatively constant over the duration of the patterns and, consequently, keep the level of inactivation of calcium channels steady. Thereby, the firing rates of regular spike patterns in afferent PCs will control the amplitude of any rebound spike that follows in the next second. The occurrence of a rebound spike is evoked by synchronized pauses in the SS trains [19], [44], which are mostly not part of the regular patterns as they belong to the tail of the ISI distribution.

In conclusion, we propose that the regular patterns, which comprise the majority of spikes in PC SS trains, can control the amplitude of subsequent timing signals by modulating the amplitude of rebound spikes in downstream DCN neurons.
